# Seasonality of service provision in hip and knee surgery: A possible contributor to waiting times? A time series analysis

**DOI:** 10.1186/1472-6963-6-22

**Published:** 2006-03-01

**Authors:** Ross EG Upshur, Rahim Moineddin, Eric J Crighton, Muhammad Mamdani

**Affiliations:** 1 Primary Care Research Unit, Sunnybrook and Women's College Health Sciences Centre, 2075 Bayview Ave, #E-349, Toronto, ON, Canada; 2 Department of Family and Community Medicine, University of Toronto, 256 McCaul Street, 2^nd ^Floor, Toronto, ON, Canada; 3 Department of Public Health Sciences, University of Toronto, McMurrich Building, 12 Queen's Park Crescent West, Toronto, ON, Canada; 4 Institute for Clinical Evaluative Sciences, 2075 Bayview Avenue, Toronto, ON, Canada; 5 Health Policy Management and Evaluation, University of Toronto, McMurrich Building, 2^nd ^Floor, 12 Queen's Park Crescent West, Toronto, ON, Canada; 6 Faculty of Pharmacy, University of Toronto, 19 Russell Street, Toronto, ON, Canada

## Abstract

**Background:**

The question of how best to reduce waiting times for health care, particularly surgical procedures such as hip and knee replacements is among the most pressing concern of the Canadian health care system. The objective of this study was to test the hypothesis that significant seasonal variation exists in the performance of hip and knee replacement surgery in the province of Ontario.

**Methods:**

We performed a retrospective, cross-sectional time series analysis examining all hip and knee replacement surgeries in people over the age of 65 in the province of Ontario, Canada between 1992 and 2002. The main outcome measure was monthly hospitalization rates per 100 000 population for all hip and knee replacements.

**Results:**

There was a marked increase in the rate of hip and knee replacement surgery over the 10-year period as well as an increasing seasonal variation in surgeries. Highly significant (Fisher Kappa = 16.05, p < 0.01; Bartlett-Kolmogorov-Smirnov Test = 0.31, p < 0.01) and strong (R^2^_Autoreg _= 0.85) seasonality was identified in the data.

**Conclusion:**

Holidays and utilization caps appear to exert a significant influence on the rate of service provision. It is expected that waiting times for hip and knee replacement could be reduced by reducing seasonal fluctuations in service provision and benchmarking services to peak delivery. The results highlight the importance of system behaviour in seasonal fluctuation of service delivery.

## Background

Waiting times for important health interventions such as hip and knee replacements, cataracts, cancer surgery, and coronary artery bypass surgery have assumed increasing prominence in publicly funded health care systems globally. In Canada, waiting times have been identified as one of the crucial challenges facing the health care system, have featured in debates during federal and provincial elections and have served as the basis for a challenge to the Canada Health Act at the level of the Supreme Court.

Prolonged wait times for crucial health interventions raise concerns about the quality of service that can be delivered by such systems. These issues figure prominently in policy debates internationally. A recent OECD report identified waiting times as a significant policy issue in 50% of all OECD countries, including among them Canada, the United Kingdom, Australia, Finland, Sweden, Denmark and the Netherlands, all of which have publicly funded health care systems [1]. Hip and knee replacements are among the highest priority conditions requiring reduction of waiting times, particularly in light of an aging population.

The determinants of waiting times are complex and likely multi-factorial in origin. A recent report in Ontario examined waiting times for hip and knee replacements and recommended that management of waiting times can be improved by more precisely understanding their determinants [2]. This report examined temporal trends in waiting times on an annualized basis showing a progressive increase in need over time. However, there is reason to suspect that annual rates alone may obscure significant seasonal influences in the delivery of care. Therefore, the objective of this study was to test the hypothesis of whether significant seasonal variation exists in the performance of hip and knee replacement surgery among a population over the age of 65 in the province of Ontario.

## Methods

We conducted a retrospective, cross-sectional time series analysis. The Canadian Institutes of Health Information Discharge Abstract Database was used to obtain monthly hospitalization data on hip and knee replacements for patients over the age of 65 beginning the first week of January 1992 to the last week of March 2002. All hip and knee replacements were extracted from the database. (Procedure codes used were as follows: ICD 9 codes: 9340, 9341, 9351 to 9353, 9359, 9365 to 9368).

This database records discharges from all inpatient hospital stays in Ontario acute-care hospitals, documenting a scrambled patient identifier, date of admission and discharge, up to 16 diagnoses as coded by the International Classification of Diseases, Ninth Revision, Clinical Modification (ICD-9-CM), and up to 10 procedures. All transfers from within one acute care hospital to another within this study group were excluded from the analysis.

Researchers using this database have found that diagnoses are coded with a high degree of accuracy [3]. There is very little missing information in the Ontario database; other province level studies have similarly found that less than 1% of the basic information on patients is missing [4-6]. The reliability of the coding of data collected by the Canadian Institute for Health Information is 74% to 96% for the ICD-9 diagnosis.

The primary outcome measure was month of procedure. The numerator consisted of all hip and knee replacements analyzed in aggregate. The procedures were summed for each month in the study period and plotted over time. The denominator was created from annual census data for residents of Ontario which were provided by Statistics Canada. Monthly population estimates were derived through linear interpolation. Using this data, monthly hospitalization rates per 100,000 population were calculated. Monthly average rates for the study period were also calculated.

To assess the statistical significance of seasonal pattern in the monthly rates of the hip and knee surgeries, spectral analysis was conducted. Spectral analysis detects periodicity in time series. The Fisher Kappa (FK) Test and the Bartlett Kolmogorov Smirnov (BKS) Test were used to detect significant departures from a random series. R^2^_Autoreg _was calculated to assess the strength of seasonality [7].

## Results

**Figure 1:**
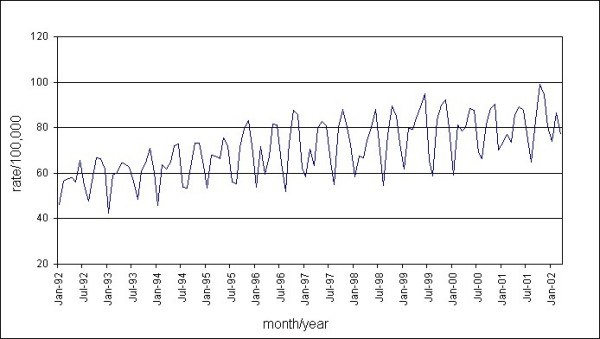
Hip and knee replacement surgery rates per 100,000 population by month and year in Ontario (1992–2002).

Table 1 shows the descriptive statistics. In the study period there were 112,168 hip and knee replacements, 43,291 in males, 68,877 in females. Figure 1 shows the rates of hip and knee replacement as expressed as monthly procedure rates per 100,000 persons. The seasonal fluctuation is apparent and the monthly peak trough ratio increases over time. The overall procedure rate increases with time, almost doubling during the period from a low of 43 procedures per 100,000 in January of 1993, to a high of approximately 100 procedures per 100,000 in October of 2001.

**Figure 2:**
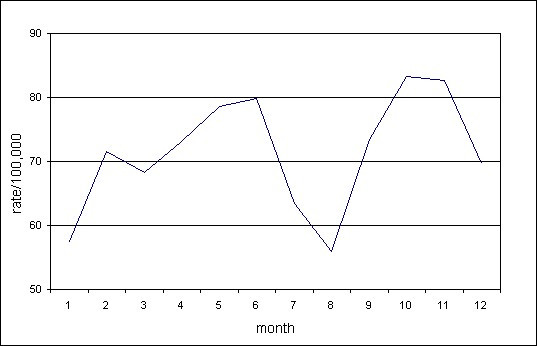
Hip and knee replacement surgery rates per 100,000 population aggregated by month in Ontario (1992–2002).

Figure 2 shows the monthly average number of procedures performed over the time period. There are marked seasonal variations in procedures. Peak capacity occurs in the fall where procedures peak at rates in excess of 83 per 100,000 population. There are substantial reductions of services corresponding to the summer months, around the Christmas and New Year's season, and to a lesser extent the March break, where procedures are seen to trough at rates as low as 56 per 100,000.

The data series was de-trended using first order differencing prior to conducting spectral analysis. Two tests for the null hypothesis that the series is strictly white noise were conducted. The Fisher Kappa (FK) Test was 16.05 (p < 0.01) and the Bartlett-Kolmogorov-Smirnov (BKS) Test was 0.31 (p < 0.01), indicating highly significant seasonality in the data. The R^2^_Autoreg _value for the series was 0.85 providing evidence of strong seasonality.

## Discussion

Our results indicate a marked increase in the rate of hip and knee replacement surgery over a 10-year period as well as an increasing seasonal variation in surgeries. The provision of services decreases during holiday times. Peak performance occurs in the fall months. Despite the increase in the provision of hip and knee replacements, the waiting times for these services have increased dramatically. Ontario data indicates that fewer 50% of eligible patients receive a joint replacement within 6 months. [2] What explains this seeming paradox? We hypothesize that seasonal fluctuation in service provision contributes to the problem.

There appears to be two major driving factors that can contribute to increasing waiting times. The segment of the population over the age of 65 has increased by approximately 23% percent over the years in this study (1,172,620 to 1,446,227). Secondly, there is marked seasonal variation in the provision of surgery indicating that the provision of surgery is not constant. Peak service occurs only in the months of October, November, and to a lesser extent, May and June. This may reflect both provider and recipient preferences not to have surgery during holiday times. Although holidays exert an influence on the provision of service, they do not affect the demand which relates to osteoarthritis, the predominant indication for surgery. This has not changed during the study period. Holidays exert a significant influence on the rate of service provision as the troughs correspond to the summer holidays, winter holidays and March school breaks.

As well, it is possible that utilization caps in the province of Ontario explain the lower rates of service provision during January-March. Until 2005, the salary of orthopedic surgeons was capped at a certain level adding financial disincentives to perform surgery for which they would not be full remunerated. The fiscal year runs from April 1 – March 31, so the January – March rates corresponds to the time that surgeons would be approaching the cap. As the caps have been lifted in 2005, their effect on rates will be testable in the years to come.

In a simple schemata, waiting lists can be understood operationally in terms of the relationship between inputs (increasing number of people needing surgery), throughputs (including seasonal fluctuation and system features), and outputs (completed surgeries). It can be hypothesized that waiting times for hip and knee replacement could be reduced with more constant supply. Eliminating seasonal fluctuations in service provision and benchmarking services to peak delivery months would increase capacity by about 400 to 500 procedures per annum. The cyclic peaks and troughs likely exacerbate waiting times as the cycle is repeated annually while the need increases steadily.

The results highlight the importance of system behaviour in seasonal fluctuation of service delivery. The OECD report identified several determinants of waiting lists including physician availability and remuneration but did not consider time of surgery as an important factor in the creation of waiting times [1]. Recent reports on waiting times for hip and knee replacements in Canada have examined geographical variation, but did not examine rates as dynamic over time despite recognizing time of surgery as a crucial component of waiting times [2]. Although it is well known that seasonal increases of health service demand are associated with viral pathogens, such as influenza, it is less well appreciated the extent to which human behaviour influences health services delivery. Attempts to rectify waiting lists without considering human behaviour will likely fail to address many issues determining waiting lists.

There are certain caveats to this analysis. First, we lack an estimate for the prevalence of need for surgery. We have assumed that the prevalence of need has increased over time in this analysis using an aging population as a proxy for surgical demand. Secondly, this analysis is limited to the province of Ontario as the unit of aggregation. Therefore, it will overlook important geographic variations within the system which may play a role in waiting list dynamics. Such factors as OR time and availability, and the number of surgeons available to perform such surgeries, are obviously important determinants of waiting times. We restricted our analysis to this population as it is the population with by far the greatest need for the procedure.

## Conclusion

The results of this study indicate the importance of understanding the temporal trends and seasonal variation of service provision. A similar analysis for other conditions plagued by waiting lists such as cataracts and cancer surgery would be informative to determine if the patterns demonstrated with hip and knee surgeries similarly exist. These results could be verified in other OECD jurisdictions.

## Competing interests

The author(s) declare that they have no competing interests.

## Authors' contributions

REGU initiated the idea for the study, interpreted the data, wrote the first draft of paper, and contributed to all subsequent drafts. RM conducted the statistical analysis, contributed to the design of the study, helped interpret the findings, and contributed to the writing of the paper. MM and EJC helped design the study, interpret the findings and contributed to the writing of the paper. All authors have read and approved the final manuscript.

## Pre-publication history

The pre-publication history for this paper can be accessed here:



## Figures and Tables

**Table 1:** Counts of knee and hip hospitalizations in Ontario: 1992–2001

**Year**	**Male**	**Female**	**Both**
1992	3051	5199	8250
1993	3398	5309	8707
1994	3592	5896	9488
1995	3966	6452	10418
1996	4192	6752	10944
1997	4482	7180	11662
1998	4681	7336	12017
1999	5227	8068	13295
2000	5274	8006	13280
2001	5428	8679	14107

**Total**	**43,291**	**68,877**	**112,168**

## References

[B1] Siciliani L, Hurst J (2003). Explaining waiting times variations for elective surgery across OECD countries.

[B2] Bourne R, DeBoer D, Hawker G, Tu J, Pinfold S, McColgan P, Laupacis A (2005). Chapter 5 Total Hip and Knee Replacement. Access to Health Services in Ontario: ICES Atlas.

[B3] Williams J, Young W (1996). Appendix I: A summary of studies on the quality of health care administrative databases in Canada. Patterns of Healthcare in Ontario The ICES Practice Atlas.

[B4] Davidson W, Molloy DW, Somers G, Bedard M (1994). Relation between physician characteristics and prescribing for elderly people in New Brunswick. CMAJ.

[B5] Rawson N, Malcolm E (1995). Validity of the recording of cholecystectomy and hysterectomy in the Saskatchewan health care datafiles.

[B6] Thiessen BQ, Wallace SM, Blackburn JL, Wilson TW, Bergman U (1990). Increased prescribing of antidepressants subsequent to beta-blocker therapy. Arch Intern Med.

[B7] Moineddin R, Upshur RE, Crighton E, Mamdani M (2003). Autoregression as a means of assessing the strength of seasonality in a time series. Popul Health Metr.

